# Neuroendocrine tumour arising inside a retro-rectal tailgut cyst: report of two cases and a review of the literature

**DOI:** 10.3332/ecancer.2011.201

**Published:** 2011-02-22

**Authors:** F Spada, G Pelosi, M Squadroni, K Lorizzo, A Farris, F de Braud, N Fazio

**Affiliations:** 1 Department of Medicine, European Institute of Oncology (IEO), Milan, Italy; 2 Department of Pathology, National Institute of Oncology (INT), Milan, Italy; 3 Division of Clinical Pharmacology and New Drugs, European Institute of Oncology, Milan, Italy; 4 Department of Oncology, University of Sassari, Sassari, Italy

## Abstract

Tailgut cysts (or retro-rectal cyst-hamartomas (RCHs)) are developmental abnormalities consisting of multiloculated cysts lined by squamous, transitional or glandular epithelium which, albeit rarely, may give rise to malignant transformations. Carcinoid tumours arising in the presacral region are extremely rare and usually benign, and only a few are described in the literature. Case 1: A 63-year-old female diagnosed as having bilateral ovarian cysts underwent surgery to remove a right adnexial mass that was histopathologically diagnosed as a well-differentiated carcinoid tumour. She is currently disease free after 18 months of follow-up. Case 2: A 41-year-old-female diagnosed with hepatic metastases and a solid pelvic mass arising from a moderately differentiated neuroendocrine carcinoma is currently alive with disease after having undergone surgical removal of the mass and several medical treatments. We here describe two different clinical histories of well- and moderately differentiated neuroendocrine tumours (NETs) arising from tailgut cysts in the prerectal space together with a review of the relevant literature.

## Background

Neuroendocrine tumours (NETs) are a relatively rare and heterogeneous group of usually indolent malignancies, and there have been sporadic reports of NETs originating in the presacral region. Tailgut cysts (or retro-rectal cyst-hamartomas, RCHs) are very uncommon, generally congenital developmental abnormalities that consist of multiloculated cysts lined with squamous, transitional or glandular epithelium, and are usually incidentally identified in adult life as a result of radiological examinations performed for other reasons. Albeit rarely, they may give rise to malignant transformations, including NETs or adenocarcinomas. Here, we describe the clinical cases of two Italian female patients with NETs which developed within a tailgut cyst.

## Methods

### Case 1

A 63-year-old woman with a negative family history of malignancy had undergone a hysterectomy because of uterine fibromas when she was 45-years-old. She also had a radiologically diagnosed slipped lumbar disc that did not require pain medication. In July 2008, she underwent radiological examinations because of increased lumbar pain and the emergence of a peri-umbilical mass. Spine magnetic resonance imaging was negative for the presence of a neoplasm and/or suspected mass, but abdominal and transvaginal ultrasonography revealed bilateral ovarian cysts. Her blood carcinoembryonic antigen and cancer antigen (CA) 125 levels were normal, but she had above-normal levels of CA 19.9 (55.60 U/ml; n.v.<33 U/ml). There were no other abnormalities.

**Figure 1:**
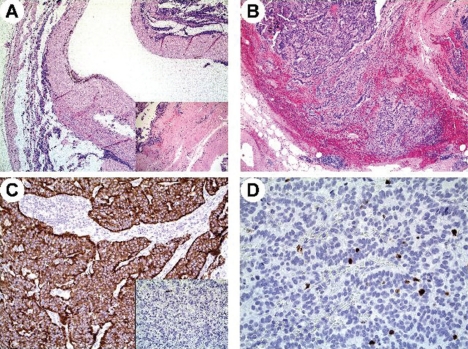
Case 1. Microscopy of the resected tumour in patient 1. Well-differentiated neuroendocrine tumour arising within a tailgut cyst exhibiting a thick, fibrous wall (A) containing residual, disarrayed smooth muscle fibres (A, inset). Tumour cells showed a trabecular pattern of growth (B), diffuse immunoreactivity for synaptophysin (C) but only focal for chromogranin A (C, inset) and a low Ki-67 labelling index (less than 2%) (D).

She underwent bilateral adnexectomy with the removal of a 58 × 35 × 43 mm cystic/solid mass located in the retroperitoneal region and loosely connected to the lower sacrum by means of fibrous attachments, leading to complete functional recovery. After being emptied, the cystic mass measured 40 × 30 × 20 mm. Macroscopically, the external wall was red and smooth, but the internal wall was multiloculated. A brown-red nodule with a diameter of 15 mm arising from the external wall was microscopically described as a NET (carcinoid) mainly with a trabecular architecture probably arising from a retro-rectal tailgut cyst. The histological material was reviewed by a pathologist (GP) at the European Institute of Oncology in Milan, who confirmed the diagnosis of a well-differentiated NET (based on the.2000 WHO classification). The trabecular architecture was compatible with a retro-rectal tail cyst origin. The immunophenotype was positive for cytokeratin AE1–AE3, synaptophysin, pancreatic polypeptide (PP), acid phosphatase and, focally, chromogranin A. The proliferation index (Ki-67) was <2%. Peritoneal washing did not reveal malignant cells. The patient received no medical therapy and after a follow-up of 25 months, is in good clinical condition with no clinical, biochemical or radiological signs of recurrence (Figure 1).

### Case 2

This is the case of a 41-year-old woman who gave birth to twins in March 2003. In April of the same year, she underwent abdominal ultrasonography because of persistent rectal pain during evacuation; a solid mass was found close to the rectum. Subsequent colonoscopy excluded the presence of inherent lesions. A chest-abdomen computed tomography (CT) scan revealed a thickened rectal wall, the solid mass and multiple liver metastases. A total body ^In^111-Octreotide scintigraphy showed several regions of uptake in the liver and hypogastric region. Blood chromogranin A and neuron-specific enolase levels were normal.

**Figure 2:**
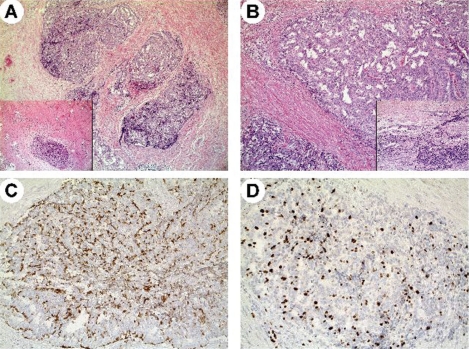
Case 2. Histological appearance of the resected tumour in patient 2. Well-differentiated neuroendocrine tumour arising within a tailgut cyst exhibiting a trabecular pattern of growth (A,B) with a residual component of disarrayed smooth muscle fibres (A, inset). Tumour cells were associated with residual glandular epithelium of tailgut cysts (B, inset) and showed faint to moderate immunoreactivity for chromogranin A (C) and a higher Ki-67 labelling index with 18% tumour cells being positive (D).

The patient underwent posterior surgery with intersphincteric and pera-sacrococcygeal excisions. The surgical specimen reviewed by a pathologist (GP) at the European Institute of Oncology in Milan indicated a moderately differentiated (Ki-67 = 18%) neuroendocrine carcinoma (on the basis of the 2000 WHO classification) associated with residual epithelial cystic material. The immunophenotype was positive for chromogranin A, synaptophysin, cytokeratin AE1–AE3, and somatostatin receptor subtype 2. On the basis of the clinical, morphological and immunohistochemical findings, a clinically malignant, presacral carcinoid with liver metastases was diagnosed (Figure 2).

Between November 2003 and April 2004, the patient received six cycles of chemotherapy (carboplatin plus etoposide) because of CT evidence of progression in the number and size of the liver lesions. Subsequently, she received peptide receptor radionuclide therapy (PRRT) from June 2004 to November 2005 because of increasing liver lesions, which led to a partial tumour response. After the appearance of pleural lesions and a solid right adnexal mass, she underwent hysterectomy and bilateral adnexectomy, and the diagnosis of a neuroendocrine carcinoma was histologically confirmed only in the left adnexa. Simultaneously, a second total body ^In^111-Octreotide scintigraphy scan revealed multiple suspected bone metastases. The patient received long-acting somatostatin analogue therapy and experienced stable disease until October 2009, when total body CT revealed the recurrence of multiple nodes. She is currently receiving PRRT with radiological stable disease.

## Discussion

Published reports of carcinoid tumours of the presacral region are rare and mainly involve direct extensions of a rectal mass [1]. They are always localized within the retro-rectal space, and are believed to originate from the remnant of the tailgut, a primitive gut temporarily present in the caudal portion of the embryo [2,3]. Their malignant transformation is very rare.

The close association of presacral carcinoid tumours with tailgut cysts suggests that both probably originate in the hindgut and remnants of the tailgut or neuroenteric cord may be the origin of tailgut or presacral space carcinoid tumours. The cysts infrequently extend from the presacral space to involve the rectal wall and rarely extend laterally from the mid-line or into the postsacral space, or laterally and anteriorly to the rectum.

To the best of our knowledge, only 28 cases of carcinoid tumours of the presacral region are described in the medical literature and of these 14 were associated with tailgut cysts. The rest were either associated with teratomas or with no other peculiar anomalies (Table 1). Although information about the clinical evolution of carcinoid tumours originating from tailgut cyst is limited, only one of them showed overt metastatic dissemination at the time of clinical diagnosis [4].

## Conclusion

Here, we describe the different behaviours of two well-differentiated NETs arising from RCHs. In the first case, despite the absence of residual epithelial material, the anatomic location of the NET and its benign behaviour make it very likely that it originated from a tailgut cyst. The second case demonstrates that not all NETs arising from tailgut cysts are benign. The second patient showed metastatic dissemination at the time of initial diagnosis and evidence of clinical recurrence over a 7-year observation period. The treatment of these malignancies may therefore vary from case to case. The time of the diagnosis, the radical nature of surgical resection and a histopathological diagnosis are the most important prognostic factors.

Although NETs arising from tailgut cysts in the retro-rectal area are extremely rare and generally benign, they should be considered in the differential diagnosis of an adnexal mass evidenced by radiological and pathological means. The behaviour of our two cases are in line with their Ki-67 index values, thus indicating this as reliable predictors in NETs.

## Figures and Tables

**Table 1:** Reported cases of carcinoid tumours arising in tailgut cysts

**Case**	**Age/gender**	**Associated anomalies**	**Follow-up**	**References**
1	NA/NA	Tailgut cyst	NA	Hood *et al*., *1988*
2	50/F	Tailgut cyst	NA	Hood *et al. 1988*
3	57/F	None	NED, 1 year	Addis *et al*., *1991*
4	18/F	Tailgut cyst	NA	Lin *et al*., *1992*
5	61/M	None	NED, 2 years	Schnee *et al*., *1994*
6	NA/F	None	NA	Edelstein *et al*., *1996*
7	19/F	Tailgut cyst	NED, 4 years	Horenstein *et al*., *1998*
8	19/F	None	NED, 3 years	Horenstein *et al*., *1998*
9	21/F	None	Local recurrence and metastases to breast	Horenstein *et al*., *1998*
10	69/F	Tailgut cyst	NA	Prasad *et al*., *1999*
11	52/M	Tailgut cyst	NA	Oyama *et al*., *2000*
12	55/F	Tailgut cyst	Synchronous liver metastases and metachronous bone metastases	M. Wöhlke *et al., 2010*
13	68/M	Tailgut cyst	NED	Mourra *et al*., *2003*
14	NA/NA	Tailgut cyst	NA	Jacob *et al.,2004*
15	41/F	Tailgut cyst	Metachronous liver and brain metastases	Song *et al., 2004*
16	49/F	Tailgut cyst	NED	Mathieu *et al.,2005*
17	40/F	Tailgut cyst	Metachronous liver and brain metastases	Lee *et al*., *2007*
18	51/F	Tailgut cyst	NED	Liang *et al*., *2008*
19	73/F	Tailgut cyst	NED	La Rosa *et al., 2010*
20	63/F	Tailgut cyst	NED	This report, *2010*
21	41/F	Tailgut cyst	Synchronous liver metastases and metachronous bone metastases	This report, *2010*
22	42/F	None	Bone/rectum Infiltration	Gorski *et al.1999*
23	51/F	None	No	Theunissen *et al.,2001*
24	72/M	None	Sacrum infiltration	Dujardin *et al., 2009*
25	40/F	None	Coccyx infiltration	Krasin *et al., 2001*

F: female; M: male; NED: no evidence of disease; NIA: not available.
